# Artificial control of the bias-voltage dependence of tunnelling-anisotropic magnetoresistance using quantization in a single-crystal ferromagnet

**DOI:** 10.1038/ncomms15387

**Published:** 2017-05-22

**Authors:** Iriya Muneta, Toshiki Kanaki, Shinobu Ohya, Masaaki Tanaka

**Affiliations:** 1 Department of Electrical Engineering and Information Systems, The University of Tokyo, 7-3-1 Hongo, Bunkyo-ku, Tokyo 113-8656, Japan; 2 Center for Spintronics Research Network (CSRN), The University of Tokyo, 7-3-1 Hongo, Bunkyo-ku, Tokyo 113-8656, Japan

## Abstract

A major issue in the development of spintronic memory devices is the reduction of the power consumption for the magnetization reversal. For this purpose, the artificial control of the magnetic anisotropy of ferromagnetic materials is of great importance. Here, we demonstrate the control of the carrier-energy dependence of the magnetic anisotropy of the density of states (DOS) using the quantum size effect in a single-crystal ferromagnetic material, GaMnAs. We show that the mainly twofold symmetry of the magnetic anisotropy of DOS, which is attributed to the impurity band, is changed to a fourfold symmetry by enhancing the quantum size effect in the valence band of the GaMnAs quantum wells. By combination with the gate electric-field control technique, our concept of the usage of the quantum size effect for the control of the magnetism will pave the way for the ultra-low-power manipulation of magnetization in future spintronic devices.

## Abstract (web summary)

Reduction of power consumption for magnetization reversal in spintronic memory devices is of great importance. Here, Muneta *et al*. report the gate electric-field assisted control of the magnetic anisotropy of the density of states using quantum size effect in GaMnAs.

The control of the magnetic anisotropy^1^,^2^,^3^,^4^,^5^,^6^,^7^ is a particularly crucial issue for the reduction of the power required for the magnetization reversal that will enable the exploitation of the full potential of the spintronic memory and logic devices, such as magnetic tunnel junctions^8^,^9^,^10^,^11^,^12^ and spin transistors^13^,^14^, that may outperform the CMOS circuits commonly used in existing computers. Meanwhile, band engineering is a useful technique to manipulate the electronic structures of materials and heterostructures using an electric field^15^, the quantum size effect^16^,^17^ and modulation doping^18^,^19^, and it is a well-established and important technology for the design of electronic devices based on semiconductors. For example, by designing quantum well structures, we can completely control the quantized energy levels or two-dimensional sub-band structures of materials as calculated using band engineering. Although band engineering was developed mainly for semiconductor electronics, it could potentially be extended to magnetism because electron spins and their motions are coupled by spin–orbit interactions. However, this point of view of the usage of band engineering is lacking^1^,^2^,^3^,^4^,^5^,^6^,^7^,^20^,^21^,^22^,^23^,^24^,^25^, and there are a wide variety of possibilities for band engineering to design or control magnetic materials and devices.

Here, we demonstrate the artificial control of the carrier energy dependence of the magnetic anisotropy of the density of states (DOS) for the first time by designing quantum well structures consisting of a single-crystal ferromagnetic thin film and semiconductor barriers and tuning the strength of the quantum size effect. We use the prototypical ferromagnetic semiconductor GaMnAs that has a band structure in which the energy regions of the valence band (VB) and impurity band (IB) overlap^26^. We find that the magnetic anisotropy of the DOS varies depending on the energy of the carriers. This phenomenon has never been reported in any ferromagnetic materials. Furthermore, we reveal that the relative strength of the magnetic anisotropy between the VB and IB, which have fourfold and twofold symmetries in the film plane, respectively, can be varied by controlling the strength of the quantum size effect induced in the VB (Fig. 1). These new findings suggest that band engineering provides the possibility of artificially designing magnetic anisotropy by controlling the electronic structures of magnetic materials. By combining this technique with the recently developed gate electric-field control technique of the carrier density and magnetization, our results will lead to ultra-low-power manipulation of magnetization in future spintronic devices.

## Results

### Samples

**Figure 2:**
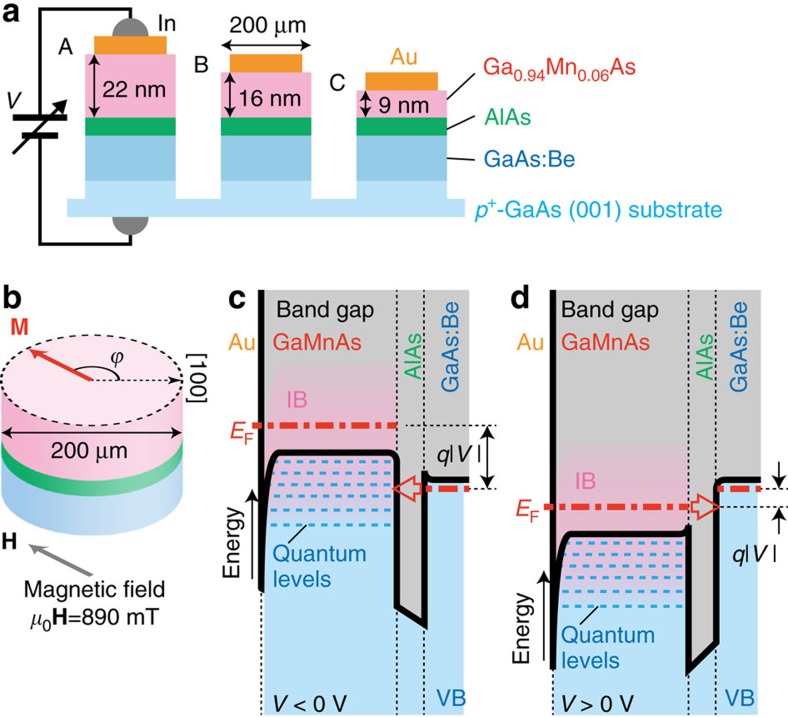
Overview of our measurements of the anisotropy. (**a**) Schematic cross-sectional structure of devices A–C used in this study. From the top surface to the bottom, an Au/GaMnAs (22, 16 and 9 nm, *T*_C_=134 K) quantum well/AlAs (5 nm)/*p*-GaAs:Be (100 nm, *p*=7 × 10^18^ cm^−3^) heterostructure is fabricated on a *p*^+^-GaAs (001) substrate. The bias voltage *V* is applied between the Au electrode and backside of the substrate. (**b**) Schematic view representing the directions of the magnetization **M** (red arrow) of the GaMnAs layer and of the magnetic field *μ*_0_|**H**|=890 mT (grey arrow) applied in our measurements. (**c**,**d**) Schematic valence band (VB) diagrams of the tunnel devices when negative (**c**) and positive (**d**) *V* are applied. The black solid and red dash-dotted lines represent the top of the VB and the quasi Fermi levels *E*_F_, respectively. The blue dash lines represent the quantized VB levels in the GaMnAs layer. These quantum levels are formed because the VB holes are confined by the surface Schottky barrier (∼1 nm) and AlAs layer^27^,^28^. The grey, blue and pink regions represent the band gap, VB and impurity band (IB), respectively. The red arrow represents the tunnel direction of the holes when *V* is applied. The character *q* represents the elementary charge.

We use a tunnel heterostructure consisting of Ga_0.94_Mn_0.06_As (25 nm, Curie temperature *T*_C_: 134 K)/AlAs (5 nm)/GaAs:Be (100 nm, hole concentration *p*=7 × 10^18^ cm^−3^) grown on a *p*^+^-GaAs (001) substrate by molecular beam epitaxy (MBE) (see Fig. 2a). We carefully etch a part of the GaMnAs layer from the surface and fabricate three tunnel diode devices with a diameter of 200 μm on the same wafer^27^,^28^ with different GaMnAs thicknesses, *d*: 22 nm (device A), 16 nm (device B) and 9 nm (device C). In the GaMnAs layer, holes are confined by the AlAs barrier and the thin depletion layer (∼1 nm) formed at the surface of the GaMnAs layer^27^,^28^, and thus the VB energy levels in the GaMnAs layer are quantized. As shown in Fig. 2b, we measure the current*–*voltage (*I–V*) characteristics at 3.5 K with a strong magnetic field *μ*_0_|**H**|=890 mT applied at an angle 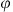 from the [100] direction in the plane so that the magnetization **M** becomes parallel to **H**, where *μ*_0_ is the vacuum permeability (see Supplementary Note 1 and Supplementary Fig. 1 for the relation between the directions of **H** and **M**). We ground the backside of the substrate and apply a bias voltage *V* to the Au top electrode. In this manner, we measure the magnetization direction dependence of the tunnel conductance at various values of *V*^29^,^30^,^31^,^32^. For these measurements, we vary *V* and 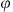 at intervals of 2 mV and 5°, respectively. As shown in Fig. 2c, when a negative *V* is applied, holes tunnel from the GaAs:Be layer to the GaMnAs layer. Because d*I*/d*V* is proportional to the DOS at the energy where tunnelling occurs, the energy dependence of the DOS below the Fermi level *E*_F_ of the GaMnAs layer is detected in negative *V*. When *V* is positive, holes tunnel from the GaMnAs layer to the GaAs:Be layer, and thus d*I*/d*V* is proportional to the DOS at *E*_F_ of GaMnAs regardless of *V* (see Fig. 2d). Our measurements provide the 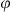 dependence of the DOS at various energies below *E*_F_ in GaMnAs.

### Analysis

**Figure 3:**
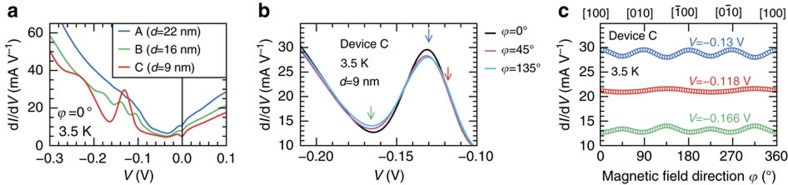
Measurement results of the tunnel transport in devices A–C. (**a**) Comparison of d*I*/d*V*-*V* curves measured on different devices when the magnetic field direction 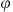 is 0°. (**b**) Comparison of three d*I*/d*V*-*V* characteristics at different 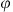. The arrows indicate the *V* values used in **c**. (**c**) The 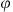 dependence of d*I*/d*V* in device C when *V* is fixed at −0.13 V (blue), −0.118 V (red) and −0.166 V (green), corresponding to the arrows shown in **b**.

**Figure 4:**
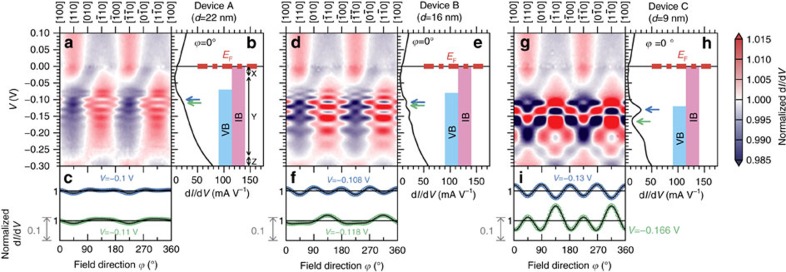
Measurement results of the magnetization-direction dependence of the tunnel transport in devices A–C. (**a**,**d**,**g**) Colour-coded maps representing the normalized d*I*/d*V* as a function of the magnetic field direction 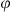 and *V*. The normalized d*I*/d*V* is defined by equation (1). The negative voltage region in device A is classified into three parts X, Y and Z by the sign of the oscillation in the normalized d*I*/d*V*-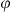 curves. (**b**,**e**,**h**) Characteristics of d*I*/d*V*-*V* at 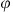
=0°. The blue and green arrows indicate *V* corresponding to the peak and dip, respectively, that are used in **c**,**f**,**i**. The blue and pink regions shown on the right side represent the valence band (VB) and impurity band (IB) regions in GaMnAs, respectively. The red dash-dotted lines represent the Fermi level *E*_F_ (*V*=0). (**c**,**f**,**i**) The normalized d*I*/d*V*-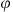 curves at *V* corresponding to the positions of the blue and green arrows in **b**,**e**,**h**, respectively. The black solid curves are the fitting curves expressed by equation (2).

The d*I*/d*V*-*V* characteristics obtained for devices A–C at 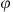
=0° show different oscillatory behaviour (see Fig. 3a); the oscillation becomes stronger as *d* decreases. Also, the *V* values of the d*I*/d*V* peaks systematically change by changing *d*, indicating that these oscillations originate from the resonant tunnelling effect^33^,^34^ induced by the quantum size effect in the VB of the GaMnAs layer^27^,^35^,^36^,^37^,^38^. (See Supplementary Fig. 3 for the *I–V* characteristics of devices A–C. More systematic data of the d^2^*I*/d*V*^2^-*V* characteristics of tunnelling devices with various GaMnAs thicknesses (*d*=7.3–23.6 nm) are described in Supplementary Note 3 and Supplementary Fig. 4.) The oscillation amplitude of d*I*/d*V* decreases by changing 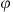 from 0° (along [100]) to 45° (along [110]) or 135° (along [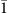
10]) (see Fig. 3b). The peak (blue arrow) value of d*I*/d*V* when 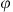
=0° is larger than that when 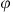
=45° or 135°, whereas the dip (green arrow) value of d*I*/d*V* when 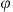
=0° is smaller than that when 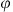
=45° or 135°. This feature can be seen in Fig. 3c as the opposite sign of the oscillation of d*I*/d*V* as a function of 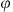 between when *V*=−0.13 V (the peak of d*I*/d*V*-*V* in Fig. 3b) and when *V*=−0.166 V (the dip of d*I*/d*V*-*V* in Fig. 3b). In Fig. 3c, the symmetry of the 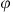 dependence of d*I*/d*V* changes depending on *V*. Here, we normalize d*I*/d*V* using


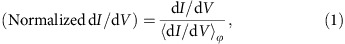


where 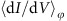 is the d*I*/d*V* value averaged over 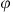 at a fixed *V*. As seen in Fig. 4a,d,g, primarily twofold symmetry along [110] is observed at negative *V* when *d*=22 nm, but fourfold symmetry along 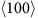 emerges as *d* decreases. At the peaks and dips of the d*I*/d*V*-*V* curves indicated by the blue and green arrows in Fig. 4b,e,h, the symmetry of the d*I*/d*V*-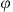 curves shown in Fig. 4c,f,i is changed from twofold to fourfold as the resonant tunnelling is enhanced by decreasing *d*. Because resonant tunnelling is induced in the VB, the enhanced fourfold symmetry is attributed to the VB. The curves in Fig. 4i are those obtained by normalizing the d*I*/d*V*-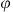 curves shown in Fig. 3c (blue and green).

We derive the symmetry components *C*_4_ (fourfold along 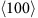), 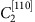 (twofold along [110]) and 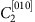 (twofold along [010]) of the normalized d*I*/d*V*-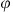 curves by fitting the following equation to the experimental normalized d*I*/d*V*-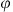 curves:


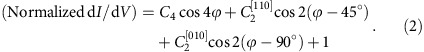


**Figure 5:**
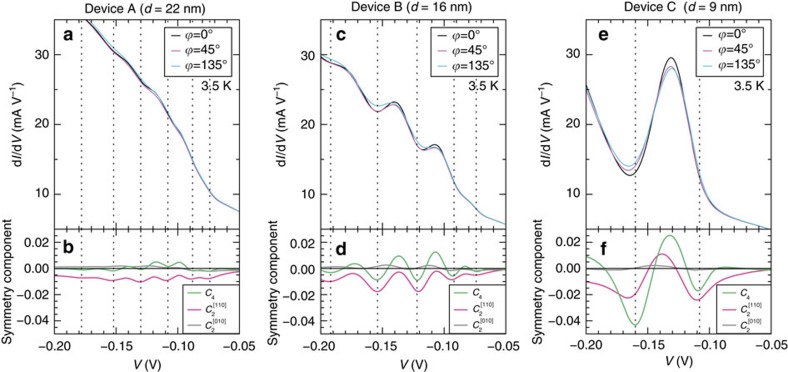
Relationship between the differential conductance and symmetry components. (**a**,**c**,**e**) The d*I*/d*V*-*V* characteristics measured at 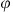
=0° (black), 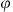
=45° (pink) and 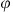
=135° (light blue) on devices A–C, respectively. (**b**,**d**,**f**) The fourfold symmetry components *C*_4_ along 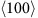 (green), twofold symmetry components 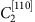 along [110] (pink) and twofold symmetry components 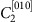 along [010] (grey) as a function of *V*. These components are obtained by fitting the curves expressed by equation (2) to the normalized d*I*/d*V*-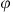 curves at each *V*. The vertical dotted lines represent the *V* at which *C*_4_ reaches dips.

The *V* dependences of *C*_4_, 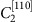 and 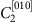 show oscillations that synchronize with the oscillations of the d*I*/d*V*-*V* curves that are induced by the resonant tunnelling (see Fig. 5). *C*_4_ is significantly enhanced as *d* is decreased and the oscillation of d*I*/d*V*-*V* is enhanced. Therefore, the oscillatory behaviour of the *V* dependence of the symmetry components is attributed to the quantization of the VB states.

We discuss the origin of the fourfold symmetry of the d*I*/d*V*-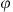 curves induced by the quantum size effect in the VB of GaMnAs. Our results mean that the strength of the quantization depends on 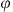 as shown in Fig. 3b; in other words, the coherence length of the VB holes depends on 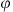. In GaMnAs, there is a weak interaction between the VB holes and Mn spin magnetic moments, indicating that the coherence length of the VB holes depends on the strength of this interaction. Thus, the fourfold symmetry of the magnetic anisotropy originates from the anisotropy of the wave function of the VB holes that are mainly composed of As 4*p* orbitals located at the lattice points having a fourfold symmetry in the film plane. This shows that the interaction between the spins and orbitals reflects the anisotropy of the wave function distribution and the direction of spins. We calculated the DOS of the VB that is weakly interacting with the magnetic moments of Mn, using the **k·p** Hamiltonian and *p*-*d* exchange Hamiltonian, and confirmed that the DOS versus 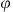 characteristic shows the fourfold symmetry (see Supplementary Note 4 and Supplementary Fig. 5).

### Magnetic anisotropy of the IB

In the region of *V*=−0.07 V–+0.1 V of the colour-coded maps shown in Fig. 4a,d,g, the normalized d*I*/d*V* as a function of 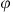 and *V* is similar in devices A–C, meaning that it is insensitive to the change in *d*, and thus the twofold symmetry along [110] observed in this region is attributed to the IB. This insensitivity to *d* agrees with the previous report^39^ that the magnetic anisotropy of magnetization in GaMnAs films at low temperature is independent of *d*. In the region of *V*>0 V, the 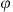 dependence of the normalized d*I*/d*V* does not depend on *V* because it always reflects the DOS at *E*_F_ in GaMnAs regardless of *V*. The twofold symmetry along [110] is also observed in the region of *V*<−0.07 V for device A (*d*=22 nm, Fig. 4a), in which the effect of the quantization of the VB holes is small. This indicates that the 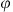 dependence of the tunnelling transport is dominated by the IB holes in the entire region of *V* when the quantization of the VB holes is weak. However, as the quantization becomes stronger, the fourfold symmetry originating from the VB emerges in the IB region, meaning that the IB and VB overlap (Fig. 4b,e,h). This finding is consistent with recent angle-resolved photoemission spectroscopy measurements of GaMnAs^26^.

**Figure 6:**
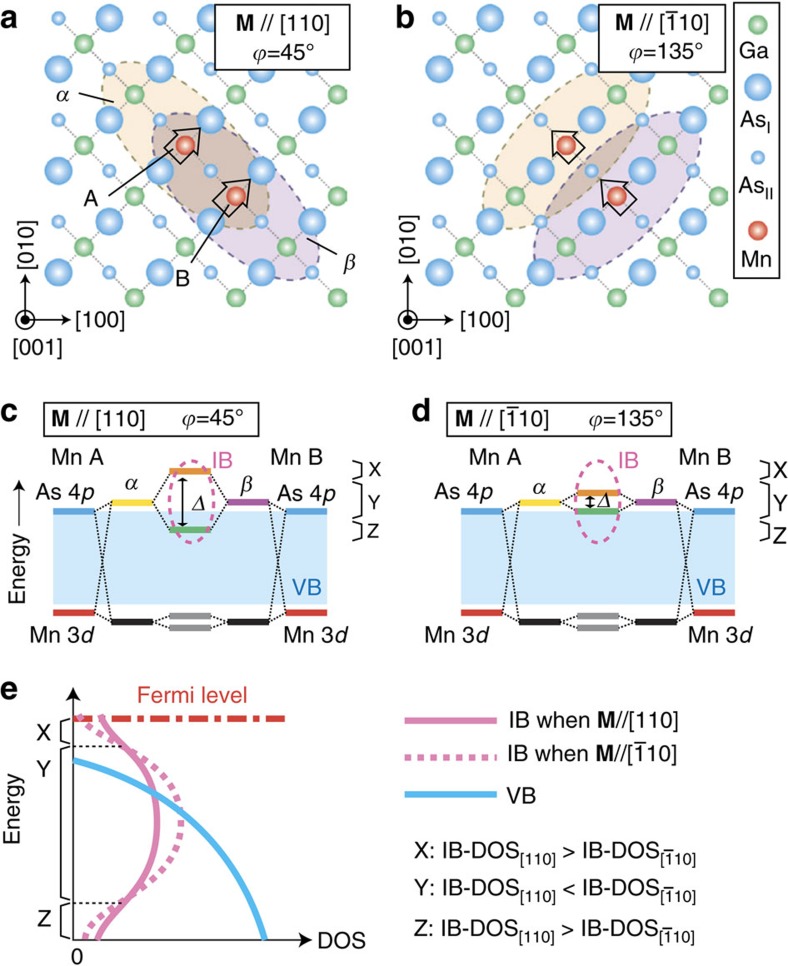
Diagrams of the electronic structures when the magnetization direction is different. (**a**,**b**) Schematic three adjoining (001) planes of GaMnAs: the upper As (As_I_), middle Ga (or Mn) and lower As (As_II_) planes when the magnetization **M** direction 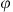 is along the [110] (**a**) and [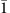
10] (**b**) directions. The ellipses schematically represent the wave functions of the impurity states *α* (yellow) and *β* (purple) originating from Mn A and B, respectively. The arrows represent the spin magnetic moments of Mn A and B. (**c**,**d**) Schematic energy-level diagrams when **M** is along the [110] (**c**) and [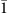
10] (**d**) directions. In each graph, the left-most (right-most) lines correspond to the 3*d* level (red) of Mn A (B) and its neighbouring As 4*p* level (blue). The yellow and purple lines represent *α* and *β*, respectively. The wavefunction overlap between *α* and *β* induces the bonding (green line) and antibonding (orange line) states with energy separation Δ. The black lines represent the localized states around Mn A and B formed by the hybridization between the 3*d* and 4*p* orbitals. The wave functions of these states overlap slightly, inducing the antibonding and bonding states with small energy separation (grey lines). (**e**) Schematic DOS around the top of the valence band (VB) in GaMnAs. The pink solid and dotted curves represent the DOS of the impurity band (IB) when **M** is along [110] and [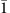
10], respectively. The blue solid curve represents the DOS of VB.

As shown in Fig. 4b, we can classify the region of *V*<0 V (corresponding to the DOS below *E*_F_ in GaMnAs) into three parts, from top to bottom: 0 V–−0.03 V (region X), −0.03 V–−0.27 V (region Y) and −0.27 V–−0.3 V (region Z). In regions X and Z, the DOS when **M** is along [110] and [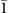

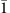
0] is larger than when **M** is along [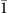
10] and [1
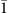
0], whereas it is smaller in region Y. We discuss the origin of this sign change of the 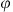 dependence of the DOS of the IB depending on the energy. A single Mn atom doped into GaAs forms an impurity state because of the inter-atomic interaction (hybridization) between the Mn 3*d* orbitals and the As 4*p* orbitals. Tang and Flatté^40^ predicted that the hybridization and spin–orbit interaction in the As 4*p* orbitals result in an antiparallel condition between the spin angular momentum of the Mn 3*d* spins and the orbital angular momentum of the hole in the impurity state. Because of this condition, the wave function of the hole in the impurity state favours extension in the direction perpendicular to the Mn 3*d* spins, and thus the distribution of the wave function depends on the direction of the Mn 3*d* spins. This behaviour is well reproduced by a tight binding method (see Supplementary Note 5 and Supplementary Fig. 6). Figure 6a,b schematically illustrates the distributions of the wave functions of the two impurity states *α* and *β* originating from the two neighbouring Mn atoms A and B located along [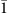
10], respectively. Here, we consider the cases in which **M** is aligned in the [110] direction (Fig. 6a) and in the [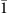
10] direction (Fig. 6b), in which the wave functions tend to extend in the [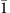
10] and [110] directions, respectively (the calculated results are shown in Supplementary Fig. 6). As shown in Fig. 6c,d, the hybridization between the Mn 3*d* (red lines) and As 4*p* (blue lines) orbitals forms *α* (yellow line) and *β* (purple line) around Mn atoms A and B, respectively. The bonding (green line) and antibonding (orange line) impurity states are formed by the wavefunction overlap between *α* and *β*, the origin of the IB^41^,^42^. As indicated in Fig. 6a,b, the wavefunction overlap between *α* and *β* is larger when **M**||[110] than that when **M**||[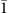
10]. Thus, the energy separation Δ between the bonding and antibonding states when **M**||[110] is larger (Fig. 6c) than that when **M**||[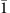
10] (Fig. 6d). The energy regions X–Z indicated in Fig. 6c,d correspond to the regions of *V* shown in Fig. 4b. When **M**||[110], the antibonding and bonding states are formed in regions X and Z, respectively (Fig. 6c). When **M**||[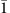
10], both antibonding and bonding states are formed in region Y (Fig. 6d). Thus, the energy dependence of the DOS of the IB differs depending on the **M** direction (that is, [110] or [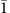
10]), as shown in Fig. 6e. In regions X and Z, the DOS when **M**||[110] is larger than that when **M**||[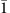
10]. In region Y, the DOS when **M**||[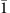
10] is larger than that when **M**||[110]. The same scenario can be applied for the Mn atoms whose distance is larger than that between the nearest Mn atoms. The twofold symmetry along the [110] axis in the DOS versus 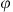 characteristic is well reproduced by a tight-binding calculation (see Supplementary Note 5 and Supplementary Fig. 7).

Above, we considered only the case in which the Mn atoms are located along [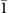
10]. Although there are several possible Mn alignments in real GaMnAs, there are reasons that only the interaction between the Mn atoms located along [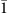
10] is important. The overlap between the wave functions of the impurity states originating from the two Mn atoms located along 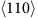 is known to be larger than that from the Mn atoms along other directions, such as 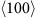 and 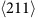
41,42. Furthermore, an anisotropic distribution of Mn atoms along [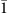
10] in GaMnAs is predicted^43^. Slightly more Mn atoms are located along [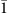
10] than along [110], and this is attributed to the direction of the Mn-As bonds on the surface during the MBE growth. This is thought to be the origin of the twofold symmetry along [110] of the d*I*/d*V*-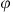 curves. Also, the anisotropic interaction between two Mn atoms and the anisotropic distribution of the Mn atoms are thought to be the reason that GaMnAs has magnetic anisotropy with the in-plane twofold symmetry along [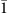
10].


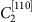
 is also slightly enhanced at the peaks and dips of the resonant oscillation in d*I*/d*V*-*V* as *d* decreases (see Fig. 5), indicating that the VB has a small twofold symmetry along [110]. The twofold symmetry of the VB is induced because the interaction between the VB holes and Mn spin magnetic moments transmits the anisotropy of the Mn distribution to the VB.

### Comparison

**Figure 7:**
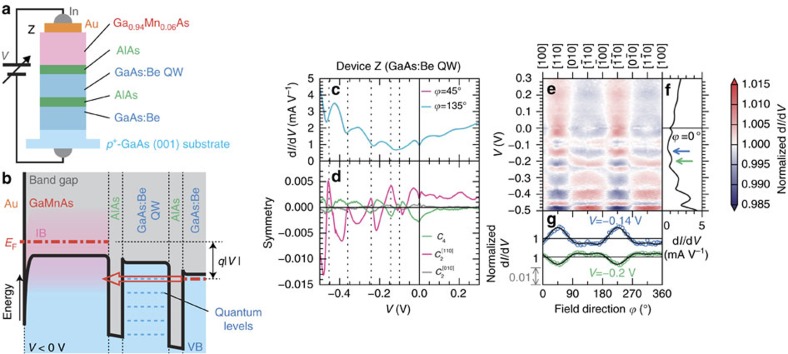
Measurement results on device Z for comparison. (**a**) Schematic cross-sectional structure of device Z used in this study. From the top surface to the bottom, an Au/Ga_0.94_Mn_0.06_As (25 nm, *T*_C_=116 K)/AlAs (6 nm)/GaAs:Be quantum well (QW) (15 nm, *p*=1 × 10^19^ cm^−3^)/AlAs (6 nm)/GaAs:Be (100 nm, *p*=3 × 10^18^ cm^−3^) heterostructure is fabricated on a *p*^+^-GaAs (001) substrate. The device Z has a nonmagnetic GaAs:Be QW and a ferromagnetic GaMnAs electrode, without a ferromagnetic QW. The bias voltage *V* is applied between an Au electrode and the backside of the substrate. (**b**) Schematic valence-band (VB) diagram of device Z when a negative *V* is applied. See Fig. 2c for the legend. (**c**) Obtained d*I*/d*V*-*V* characteristics with the magnetic field directions 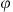
=45° (pink) and 135° (light blue) at 3.5 K. These two curves are nearly completely overlapping. (**d**) The fourfold symmetry component *C*_4_ along 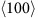 (green), twofold symmetry component 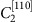 along [110] (pink) and twofold symmetry component 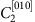 along [010] (grey) as a function of *V*. These components are obtained by fitting the curves expressed by equation (2) to the normalized d*I*/d*V*-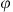 curves at each *V*. The vertical dotted lines in (**c**,**d**) represent the *V* at which *C*_4_ reaches a dip. (**e**) Colour-coded map representing the normalized d*I*/d*V* as a function of 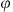 and *V*. (**f**) Characteristic of d*I*/d*V*-*V* at 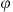
=0°. The blue and green arrows indicate the *V* of the dip and peak that are used in **g**. (**g**) Normalized d*I*/d*V*-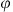 curves at *V*=−0.14 V (blue) and *V*=−0.2 V (green) indicated by the blue and green arrows in **f**, respectively. The black solid curves are obtained by the fitting.

To verify that the fourfold symmetry of the magnetic anisotropy of DOS is induced by the quantization in the GaMnAs quantum well (QW), we compare devices A–C with device Z that consists of GaMnAs (25 nm, *T*_C_ 116 K)/AlAs (6 nm)/GaAs:Be QW (15 nm)/AlAs (6 nm)/GaAs:Be (100 nm) grown on a *p*^+^-GaAs (001) substrate (see Fig. 7a,b), in which the ferromagnetic layer and the QW layer are separated and the quantum size effect does not occur in the ferromagnetic layer. The surface GaMnAs layer is thick enough to prevent the quantum size effect in this layer. We perform the same measurement on device Z. The obtained d*I*/d*V*-*V* characteristics oscillate because of the quantum size effect in the nonmagnetic GaAs:Be QW (see Fig. 7c). The *V* dependence of the normalized d*I*/d*V*-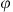 curves shown in Fig. 7e reflects the energy dependence of the magnetic anisotropy of the DOS of the GaMnAs electrode and exhibits an oscillatory behaviour that is attributed to the oscillation of the d*I*/d*V*-*V* curves induced by the resonant tunnelling effect in the GaAs:Be QW (see Fig. 7f). Similar to devices A–C, *C*_4_, 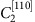 and 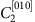 oscillate as a function of *V*, synchronizing with the oscillation of d*I*/d*V*-*V* (Fig. 7c,d); however, the symmetry of the 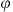 dependence of the normalized d*I*/d*V* is mainly twofold, reflecting the magnetic anisotropy of the GaMnAs top electrode (see Fig. 7d,e,g), that offers a remarkable contrast to the results of devices A–C that the quantum size effect in the GaMnAs QW layer enhances *C*_4_ (fourfold symmetry). This contrast provides evidence that the magnetic anisotropy of the DOS changes by enhancing the quantum size effect in the GaMnAs QW in devices A–C. Between the different *V* values indicated by the green and blue arrows in Fig. 7f, the symmetry of the d*I*/d*V*-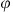 curves shows an opposite sign (Fig. 7g) that is induced by the small shift of the peak *V* of the d*I*/d*V*-*V* curves (see Supplementary Note 2 and Supplementary Fig. 2 for details).

It should be noted that surface states, if any, may have different anisotropy from the bulk or quantum well states. However, the surface of GaMnAs is depleted and does not induce the carrier-mediated ferromagnetism. This means that the surface does not couple with the magnetization of the GaMnAs layer beneath the surface. Thus, the DOS of the nonmagnetic surface does not depend on the direction of the magnetization of the GaMnAs layer.

The magnetic anisotropy of magnetization in ferromagnetic materials reflects that of the DOS at *E*_F_^30^. Our study indicates that the magnetic anisotropy of DOS depends on the carrier energy (applied voltage) and can be controlled by band engineering. Combining our results with the electric-field gating technique to tune the *E*_F_ position will provide a new method to manipulate the magnetization direction by controlling the magnetic anisotropy with an ultra-low power. This method will be useful for the development of nonvolatile spin devices using magnetization in the future.

## Methods

### Sample preparation

We grew a Ga_0.94_Mn_0.06_As (25 nm)/AlAs (5 nm)/GaAs:Be (100 nm, *p*=7 × 10^18^ cm^−3^) tunnel heterostructure by MBE for the fabrication of devices A–C. The GaAs:Be, AlAs and GaMnAs layers were grown at 550, 530 and 210 °C, respectively. We grew a Ga_0.94_Mn_0.06_As (25 nm)/AlAs (6 nm)/GaAs:Be QW (15 nm, *p*=1 × 10^19^ cm^−3^)/AlAs (6 nm)/GaAs:Be (100 nm, *p*=3 × 10^18^ cm^−3^) tunnel heterostructure for device Z. The GaAs:Be electrode, AlAs, GaAs:Be QW and GaMnAs layers were grown at 570, 530, 400 and 210 °C, respectively. The growth rates of GaAs, AlAs and GaMnAs were 500 nm h^−1^.

After the growth, we annealed the samples in air at 180 °C for 38 h for devices A–C and 20 h for device Z to improve the crystallinity and *T*_C_ of the GaMnAs layers^44^,^45^,^46^,^47^,^48^. We estimated the *T*_C_ of the GaMnAs layers by measuring the magnetic circular dichroism (MCD) on the samples and analysed the Arrott plots derived from the MCD-*μ*_0_|**H**| curves at various temperatures. The estimated *T*_C_ is 134 K for devices A–C and 116 K for device Z.

We fabricated tunnel diode devices on the wafer after growth. In our process, we used chemical wet etching with an acid solution composed of phosphoric acid, hydrogen peroxide and water. For devices A–C, we sank the wafer vertically into the etching liquid so that the thickness of the GaMnAs layer changes from 0 to 25 nm in the same wafer. Then, we made circular mesa devices with a diameter of 200 μm on the wafer by chemical wet etching for devices A–C and Z. We then coated a negative insulating resist on the wafers for the passivation of the surfaces and opened contact holes with a diameter of 180 μm on the mesa devices. Then, we deposited Au on the wafers and fabricated contact pads on them.

We carried out the device process, especially the procedure from the surface etching to the Au evaporation, very quickly to minimize the oxidation of the surface GaMnAs layer. Indeed, the resonant levels systematically change with the change in the thickness *d* of the GaMnAs layer as shown in Supplementary Fig. 4. Also, similar results have been observed in tunnelling devices with GaMnAs fabricated by similar methods^27^,^49^. Furthermore, the 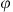 dependence of d*I*/d*V* around 0 V and in the positive *V* region is similar among samples A–C as shown in Fig. 4a,d,g. Therefore, extrinsic effects induced by the device process are negligible.

### Data procedure

We obtained the derivative of the *I–V* characteristics numerically using the Savitzky–Golay filter. We used seven data points to obtain the derivative at a single point.

In our fitting, we determined the fitting parameters by the modified Levenberg–Marquardt least squares method.

### Data availability

The data that support the findings of this study are included in Supplementary Information, and other data are available from the corresponding authors on request.

## Additional information

**How to cite this article**: Muneta, I. *et al*. Artificial control of the bias-voltage dependence of tunnelling-anisotropic magnetoresistance using quantization in a single-crystal ferromagnet. *Nat. Commun.* **8,** 15387 doi: 10.1038/ncomms15387 (2017).

**Publisher's note**: Springer Nature remains neutral with regard to jurisdictional claims in published maps and institutional affiliations.

## Supplementary Material

Supplementary Information Supplementary Figures, Supplementary Notes and Supplementary References

## Figures and Tables

**Figure 1:**
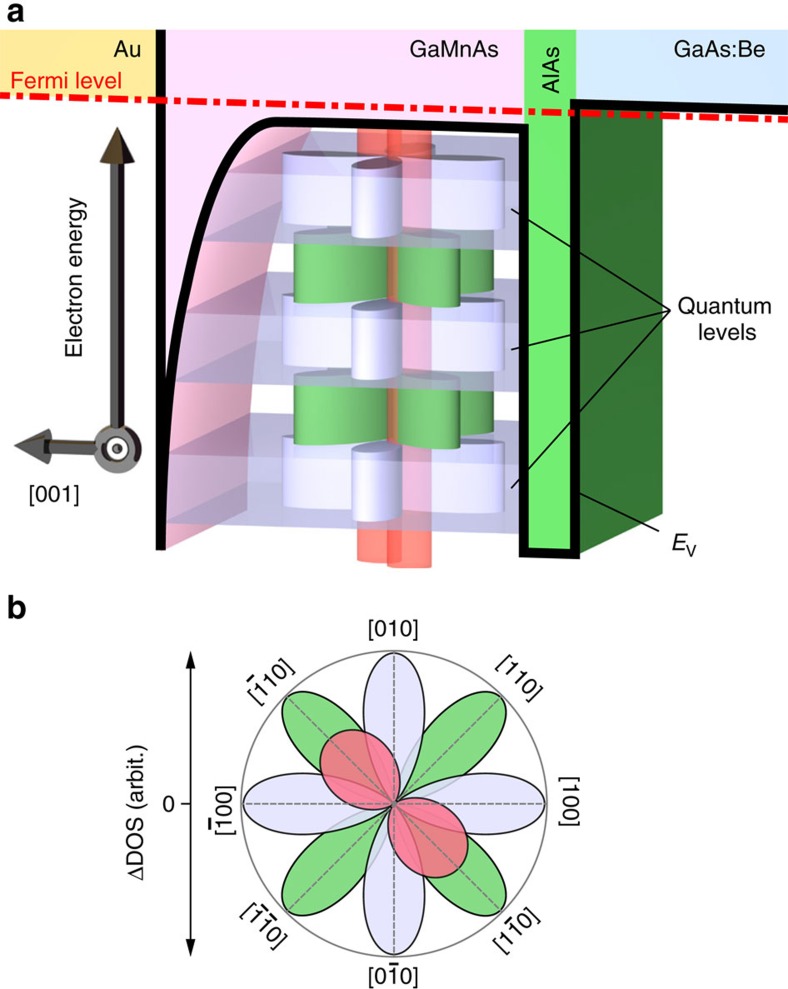
Schematic valence band diagram representing our findings. The mainly twofold symmetry of the magnetic anisotropy of the density of states (DOS), which is attributed to the impurity band, is changed to a fourfold symmetry by enhancing the quantum size effect in the valence band in GaMnAs. (**a**) The sample structure examined here is an Au/GaMnAs (22, 16 and 9 nm) quantum well/AlAs (5 nm)/*p*-GaAs:Be (100 nm)/*p*^+^-GaAs (001) substrate, from the top surface to the bottom. The yellow, pink, green and blue regions correspond to the Au, GaMnAs, AlAs and GaAs:Be layers, respectively. The red dash-dotted line and black solid curve represent the Fermi level and top energy of the valence band *E*_v_ of each layer, respectively. Three blue horizontal plates represent the quantum levels of the GaMnAs valence band. The twofold symmetry (red) and fourfold symmetry (light blue and green) represent the polar plots of the magnetic field direction dependence of the ΔDOS of the impurity band and quantized valence band, respectively. Here, ΔDOS is the change in the DOS from the minimum and the azimuth is the in-plane magnetization direction angle 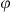 of the GaMnAs layer. These are based on the polar plots of the d*I*/d*V*-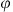 curves shown in Fig. 4c,i. At the on-resonant states (blue plates), the phase of the fourfold symmetry (light blue) is opposite to that at the off-resonant states (green). The twofold symmetry, which dominates the entire energy region, is changed to fourfold symmetry as the GaMnAs quantum well thickness is decreased and the quantum size effect is enhanced. (**b**) Magnetic field direction dependence of DOS in the (001) plane. The curves filled with blue and green represent the fourfold symmetries corresponding to the valence band on-resonance and off-resonance, respectively. The red-filled curve represents the twofold symmetry corresponding to the impurity band.
