# Supplementary material for: Artificial control of the bias-voltage dependence of tunnelling-anisotropic magnetoresistance using quantization in a single-crystal ferromagnet
Source: Nat Commun. 2017 May 22;8:15387. doi: 10.1038/ncomms15387 (PMC5458150; doi:10.1038/ncomms15387)
Supplement: Supplementary Information — Supplementary Figures, Supplementary Notes and Supplementary References [file ncomms15387-s1.pdf]

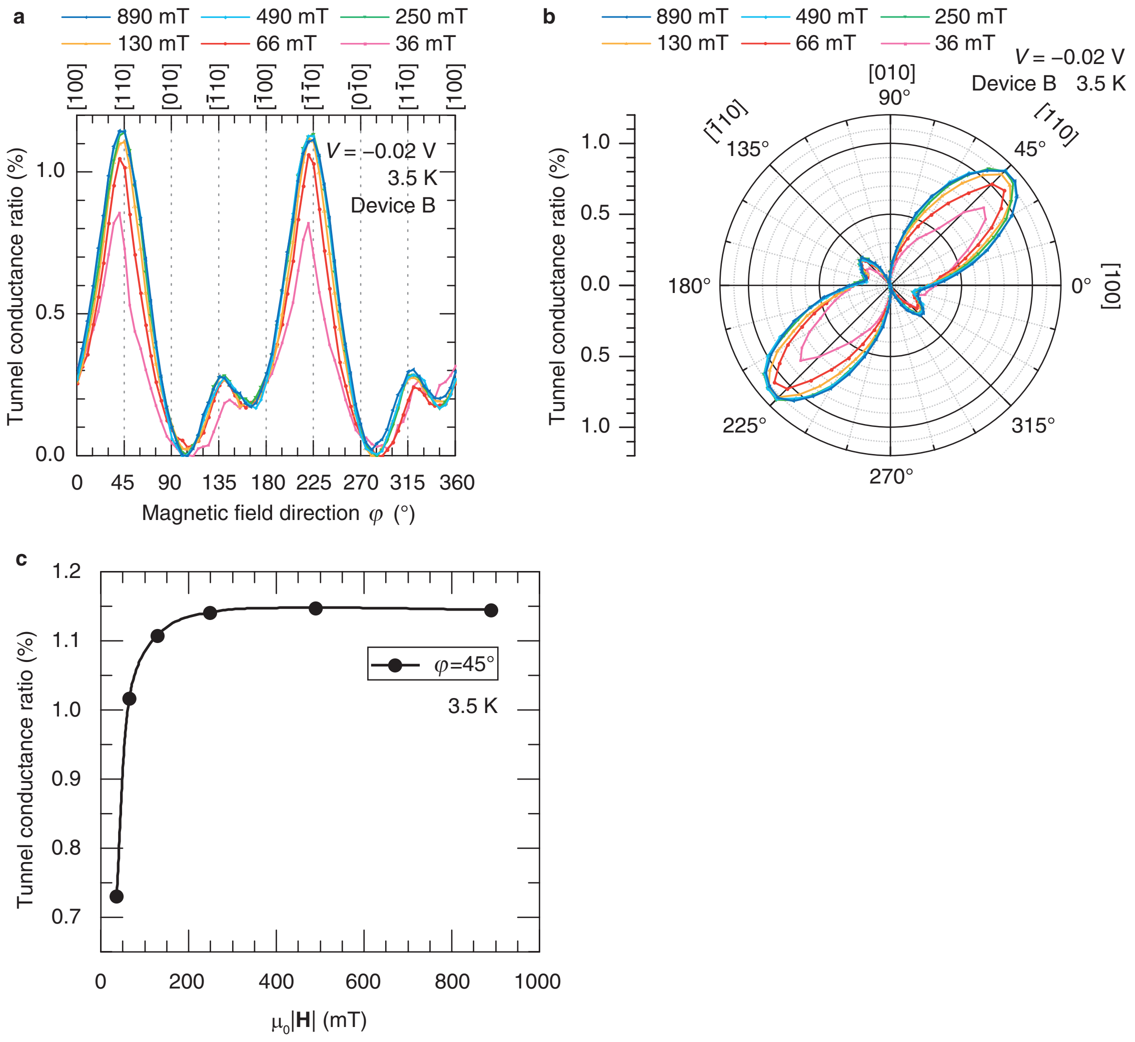

Supplementary Figure 1 | Magnetic field direction (φ) dependence of the tunnel conductance. **a**, Tunnel conductance ratio, which is defined as the ratio of the tunnel conductance to the minimum value of the tunnel conductance in the φ dependence, as a function of φ is measured at $V = -0.02$ V at 3.5 K on device B. The applied magnetic field $\mu_0|\mathbf{H}|$ is changed from 36 mT to 890 mT. **b**, Polar plot of the tunnel conductance ratio as a function of φ, corresponding to **a**. **c**, Tunnel conductance ratio as a function of $\mu_0|\mathbf{H}|$ at $\varphi = 45°$.

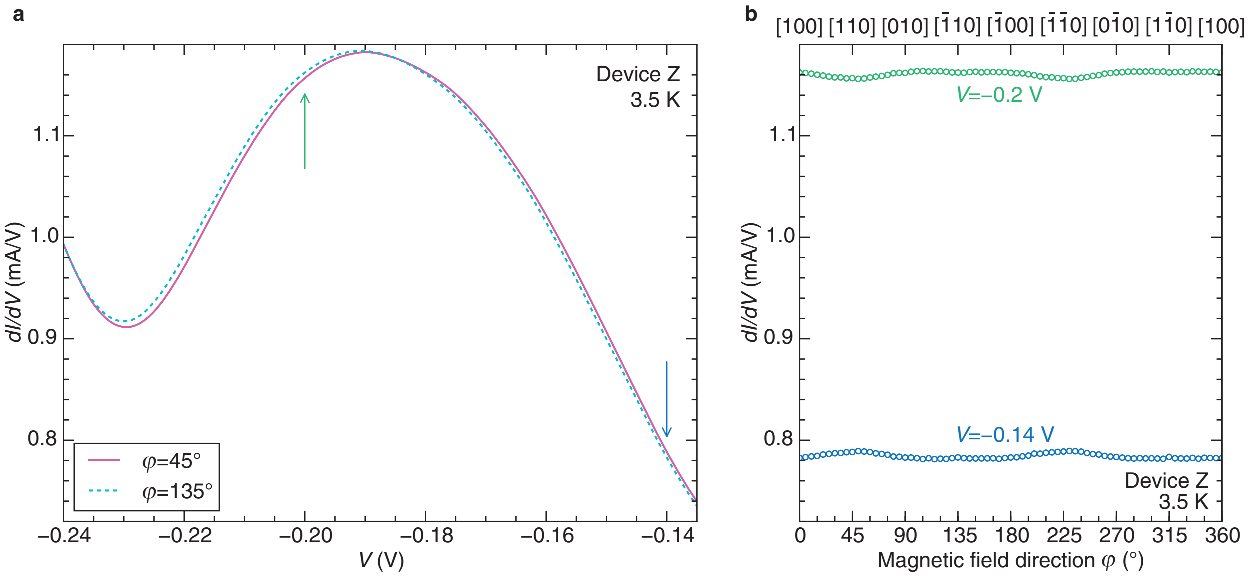

Supplementary Figure 2 | dI/dV-V characteristics around the resonant peak and the φ dependence of dI/dV in device Z. **a**, Comparison of two dI/dV-V characteristics between φ = 45° (pink) and φ = 135° (blue). The arrows indicate the V values used in **b**. **b**, φ dependence of dI/dV in device Z when V is fixed at −0.14 V (blue) and −0.2 V (green), which correspond to the arrows shown in **a**.

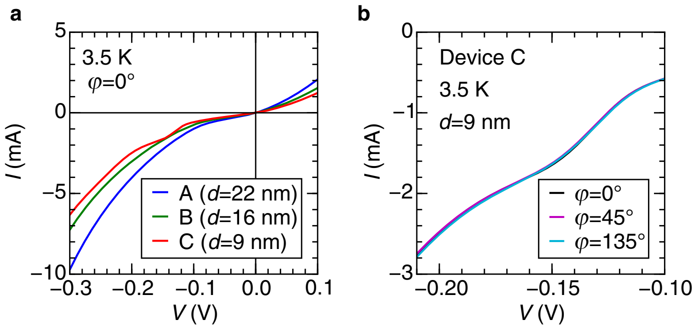

Supplementary Figure 3 | *I*-*V* characteristics at 3.5 K corresponding to Fig. 3a,b. (a) Blue, green and red curves are the *I*-*V* characteristics of devices A-C, respectively. The magnetic field direction φ is 0°. (b) Black, purple and light green curves are the *I*-*V* characteristics of device C at φ=0°, φ=45° and φ=135°, respectively.

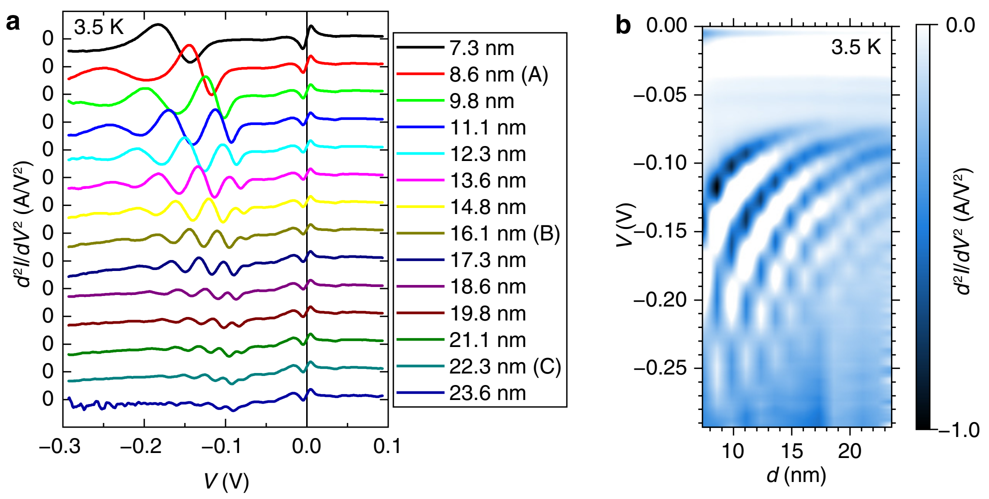

Supplementary Figure 4 | d^2I/dV^2-V curves of tunnelling devices with various GaMnAs thicknesses. The GaMnAs thicknesses d are ranging from 7.3 nm to 23.6 nm. The measurements are done at 3.5 K. **a**, Individual curves of the d^2I/dV^2-V characteristics. **b**, Colour-coded map of the d^2I/dV^2 intensity as a function of V and d.

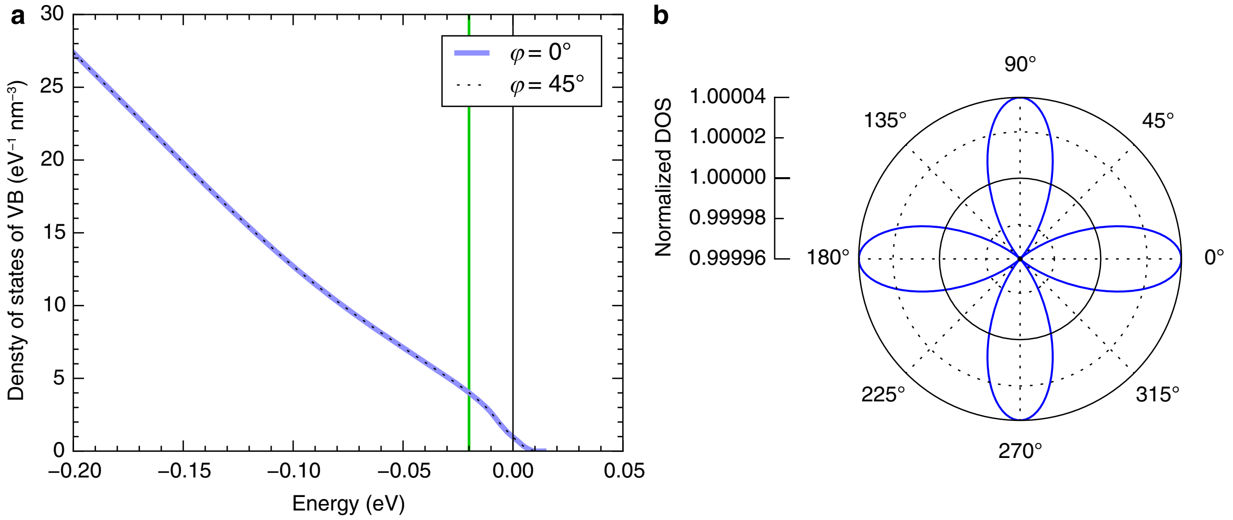

Supplementary Figure 5 | Calculated density of states of the valence band (VB). a,**b**, The DOS of VB calculated by the **k**·**p** and *p*-*d* exchange model as a function of the electron energy (**a**) and as a function of the **M** direction φ (**b**). The blue solid and black dotted curves represent VB DOS when **M** || [100] (φ = 0°) and **M** || [110] (φ = 45°), respectively. The φ dependence of the DOS of VB shown by the blue curves in **b** is the one at −0.02 eV (the vertical green line in **a**). The DOS of VB is the total DOS of the heavy and light hole bands. Here, the top of the unperturbed VB is 0 eV.

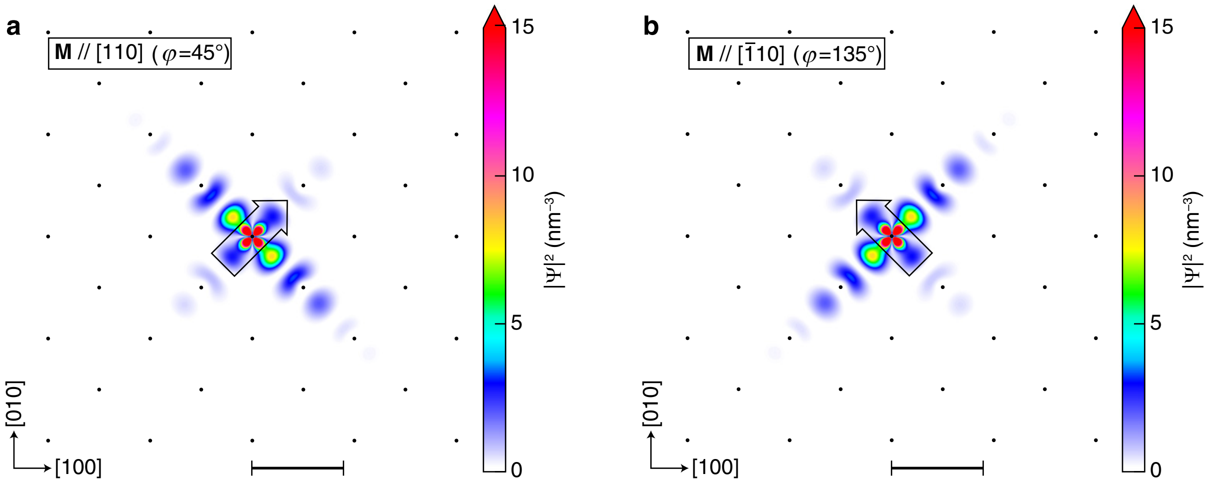

Supplementary Figure 6 | Calculated probability density of a hole in the impurity band (IB) on a (001) plane. **a**,**b**, The Mn 3*d* spins are pointing to [110] (φ=45°) (**a**) and [$\bar{1}$10] (φ=135°) (**b**). One Mn atom is doped at the center. The black dots represent the Ga sites on the plane. The arrows represent the direction of the Mn spins. The scale bar represents 0.5 nm.

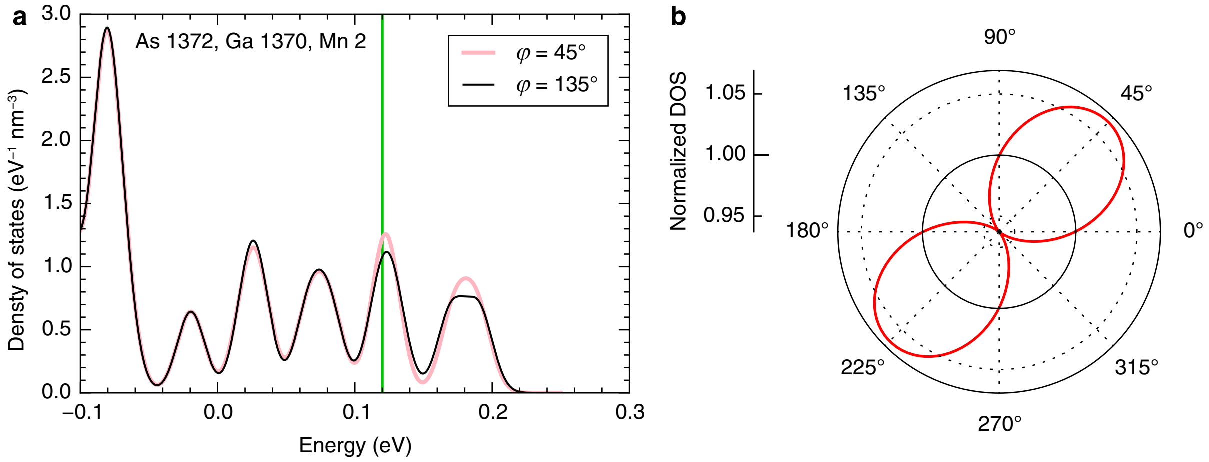

Supplementary Figure 7 | Calculated density of states of the impurity band (IB). a,b, The density of states calculated by the tight-binding method as a function of the electron energy (**a**) and as a function of the in-plane magnetization **M** direction φ (**b**). The pink and black solid lines represent DOS when **M** || [110] (φ = 45°) and **M** || [$\bar{1}$10] (φ = 135°), respectively (**a**). The φ dependence of the DOS of IB shown by the red curves in **b** is the one at 0.12 eV (the vertical green line in **a**). The top of the unperturbed VB is 0 eV.

Supplementary Note 1. Relation between the directions of the magnetic field and magnetization

In this study, we measured the magnetization (**M**) direction dependence of the tunnel conductance. We controlled the **M** direction by changing the direction of the applied magnetic field $\mu_0\mathbf{H}$. Here, μ_0 is the vacuum permeability. To check that $\mu_0|\mathbf{H}| = 890$ mT is large enough to make **M** parallel to $\mu_0\mathbf{H}$, we measured the $\mu_0\mathbf{H}$ direction (φ) dependence of the tunnel conductance with changing $\mu_0|\mathbf{H}|$ in device B. Supplementary Fig. 1a,b shows the tunnel conductance ratio as a function of φ at $V = -0.02$ V. The applied $\mu_0|\mathbf{H}|$ is changed from 36 mT to 890 mT. The tunnel conductance ratio is defined as the ratio of the tunnel conductance to the minimum value of the tunnel conductance. At $\mu_0|\mathbf{H}| = 36$ mT, the tunnel conductance ratio around [110] and $[\bar{1}\bar{1}0]$ is smaller than that when $\mu_0|\mathbf{H}| > 36$ mT. Since the [110] direction is a hard axis in GaMnAs, **M** is *not* thought to be parallel to $\mu_0\mathbf{H}$ at $\mu_0|\mathbf{H}| = 36$ mT. As $\mu_0|\mathbf{H}|$ increases, the tunnel conductance ratio around [110] and $[\bar{1}\bar{1}0]$ increases, and saturates at $\mu_0|\mathbf{H}| = 250$ mT (see Supplementary Fig. 1c). Also, as shown in the polar plot in Supplementary Fig. 1b, the tunnel conductance ratio is saturated when $\mu_0|\mathbf{H}| \geq 250$ mT. Therefore, at $\mu_0|\mathbf{H}| = 890$ mT, **M** is parallel to $\mu_0\mathbf{H}$.

Supplementary Note 2. Opposite sign of the symmetry of the dI/dV-φ curves in device Z

The structure of device Z is GaMnAs / AlAs / GaAs:Be quantum well / AlAs / GaAs:Be from the top to the bottom. The V values of the resonant peaks in the dI/dV-V curves measured on device Z are slightly different between $\varphi = 45°$ and $\varphi = 135°$ (see Supplementary Fig. 2a), which is attributed to the φ dependence of the DOS of the GaMnAs layer. Because of the shift of the peak V, the symmetry of the dI/dV-φ curves shown in Supplementary Fig. 2b shows an opposite sign between the different V below and above the resonant peak indicated by the green and blue arrows in Supplementary Fig. 2a. The shift of the resonant peak V because of the change of the **M** direction was reported in Ref. 1.

Supplementary Note 3. Evidence of the resonant tunnelling induced in the GaMnAs quantum well (QW)

The most important evidence of the resonant tunnelling is the GaMnAs QW thickness d dependence of the d^2I/dV^2-V characteristics, which are measured on the devices with various d (7.3 nm – 23.6 nm) fabricated on the same wafer (including devices A-C). Supplementary Fig. 4a,b show d^2I/dV^2-V characteristics at various d values, in which we see peaks and dips (oscillations), and as d is decreased, their positions are shifted to lower (larger minus) bias. This d-dependence clearly indicates that the oscillations are caused by resonant tunnelling through the quantized VB energy states in the GaMnAs layers, *not* by charge trapping/de-trapping at any impurity energy levels.

As shown in Supplementary Fig. 4a, a strong oscillation is observed in the device of d = 7.3 nm. As d is increased, the oscillation amplitude is decreased. Also, the dip voltages of d^2I/dV^2 becomes higher (becomes close to 0 V) and they converge on −0.07 V. These results are well explained by the d-dependence of the quantized energy levels of the VB in the GaMnAs layers, and by weakening of the quantum size effect with the increase of d. These are similar to the results of our previous studies [2, 3].

Moreover, we have fabricated the GaMnAs (30 nm) / AlAs / GaAs:Be structure, where the GaMnAs layer is thick enough to prevent the surface quantization. Although a small triangular potential is formed at the AlAs / GaAs:Be interface, the measured d^2I/dV^2-V curve does not show any oscillation. This result indicates that no oscillation is observed when there is no quantum size effect in the GaMnAs layer (d > 30 nm) [4].

Supplementary Note 4. Four-fold symmetry of the M direction dependence of VB DOS

Recent experiments have shown that the spin splitting of the valence band (VB) in GaMnAs is less than several meV [2, 5]. Thus, the VB in GaMnAs is appropriately represented by Luttinger-Kohn 6×6 **k·p** Hamiltonian with spin-orbit interaction [6] and *p-d* exchange Hamiltonian with small exchange splitting B_{G} [7],

$$H_{\mathrm{VB}} = H_{kp} + H_{pd}. \tag{1}$$

The Luttinger parameters γ_1, γ_2 and γ_3 are 6.98, 2.06 and 2.93, respectively [8]. The spin-orbit splitting, B_{G}, and the lattice constant are 0.341 eV [8], 0.003 eV and 0.5653 nm [8], respectively. As shown in Supplementary Fig. 5, the calculated DOS of the VB slightly depends on the **M** direction, and it shows the four-fold symmetry.

Supplementary Note 5. Two-fold symmetry of the M direction dependence of the IB DOS

Hamiltonian. In the calculation of the impurity band (IB) DOS, we use the Slater-Koster nearest-neighbor sp^3 tight-binding method with spin-orbit interaction [9, 10]. Two supercells are used in the calculation. One consists of 1372 As atoms, 1371 Ga atoms and 1 centered Mn atom, and the other consists of 1372 As atoms, 1370 Ga atoms and 2 Mn atoms with 0.4 nm distance along $[\bar{1}10]$. The lattice structure of the supercells is the zinc-blende structure consisting of the anion face-centered cubic (fcc) and the cation fcc, which is formed by shifting the anion fcc to the $[11\bar{1}]$ direction. We set periodic boundary conditions to the supercells. The basis functions in the system are 8 outermost sp^3 orbitals of all As and Ga atoms and 10 $3d$ orbitals of all Mn atoms. The calculation parameters are the same as those for GaAs used in ref. [10], except for the on-site potential of the Mn $3d$ orbitals and the $pd\sigma$ value between the Mn and As atoms. The on-site potential of the Mn $3d$ orbitals is 4 eV below that of the As $4p$ orbitals for the up spins. Meanwhile, the down spins of the Mn $3d$ orbitals is set to 100 eV above the As $4p$ orbitals to incorporate the electron correlation in the Mn $3d$ shell and to eliminate the effect of the interaction of the down spin electrons. The $pd\sigma$ value between the Mn and As atoms is 0.55 eV so that the impurity levels are induced at ~0.1 eV above the VB maximum. According to Harrison's formalism [11], the $pd\pi$ value is obtained from $(pd\pi) = (pd\sigma)/2.17$.

Probability density distribution. Three impurity levels at ~0.1 eV above the VB maximum are obtained by solving the eigenvalue problem of the supercell with one Mn atom. One Mn atom provides one hole, which occupies the highest impurity level. The wave function of the IB hole is represented by the linear combination of the basis functions

$$\Psi(\mathbf{r}) = \sum_i C_i \, \phi_i(\mathbf{r}), \tag{2}$$

where i, C_i and ϕ_i are the index of the basis functions, i-th element of the eigenvector and i-th basis function, respectively. To display the spatial distribution of the probability density $|\Psi|^2$, we use Slater-type orbitals [12] with Clementi's effective nuclear charge [13]. The calculated probability density of the IB hole in the supercell with one Mn atom distributes around the Mn atom within 1 nm (Supplementary Fig.6). The distribution along [110] and $[\bar{1}10]$ is not equivalent, and the difference depends on the direction of the Mn $3d$ spins. The probability density distributed along the $[\bar{1}10]$ axis (along the [110] axis) is higher when the Mn $3d$ spins are pointing to the [110] axis (pointing to the $[\bar{1}10]$ axis). This result is similar to the previous study by Tang and Flatté [14].

DOS of the supercell with two Mn atoms. Because the probability density of the IB hole is anisotropic, the wave function overlap between the IB holes of two neighboring Mn atoms depends on the direction of the two atoms arrangement [15–17]. In addition, the probability density distribution is changed by rotating the Mn 3*d* spins. Thus, the interaction between the two IB holes is changed by rotating the Mn 3*d* spins, which results in the change of the impurity level and IB DOS. The DOS of the supercell with two Mn atoms with 0.4 nm distance along $[\bar{1}10]$ is calculated by summing the normal distribution around the energy levels

$$D(E) = \frac{1}{V}\sum_i \frac{1}{\sqrt{2\pi\sigma^2}} \exp\left[-\frac{(E-E_i)^2}{2\sigma^2}\right], \tag{3}$$

where, i, E_i and V are the index of the eigenstates of the Hamiltonian matrix, i-th eigen energy and the volume of the super cell. The standard deviation σ is 0.01 eV. As shown in Supplementary Fig. 7a, DOS is induced above the VB maximum (0 eV), which is the DOS of IB. The IB DOS above the VB maximum is different between the two cases: the Mn 3*d* spins are pointing to [110] (φ=45°) and $[\bar{1}10]$ (φ=135°). The spin direction dependence of IB DOS shows two-fold symmetry along [110] (Supplementary Fig. 7b).

Supplementary References

[1] Tran, M. *et al.* Magnetization-controlled conductance in (Ga,Mn)As-based resonant tunneling devices. *Appl. Phys. Lett.* **95**, 172101 (2009).

[2] Ohya, S., Takata, K. & Tanaka, M. Nearly non-magnetic valence band of the ferromagnetic semiconductor GaMnAs. *Nature Phys.* **7**, 342 (2011).

[3] Muneta, I., Ohya, S., Terada, H. & Tanaka, M. Sudden restoration of the band ordering associated with the ferromagnetic phase transition in a semiconductor. *Nature Commun.* **7**, 12013 (2016).

[4] Ohya, S., Takata, K., Muneta, I., Hai, P. N. & Tanaka, M. Comment on "Reconciling results of tunnelling experiments on (Ga, Mn) As" arXiv: 1102.3267 v2 by Dietl and Sztenkiel. Preprint at http://arxiv.org/abs/1102.4459 (2011).

[5] Kobayashi, M. *et al.* Unveiling the impurity band induced ferromagnetism in the magnetic semiconductor (Ga,Mn)As. *Phys. Rev. B* **89**, 205204 (2014).

[6] Luttinger, J. M. & Kohn, W. Motion of electrons and holes in perturbed periodic fields. *Phys. Rev.* **97**, 869–883 (1955).

[7] Dietl, T., Ohno, H. & Matsukura, F. Hole-mediated ferromagnetism in tetrahedrally coordinated semiconductors. *Phys. Rev. B* **63**, 195205 (2001).

[8] Vurgaftman, I., Meyer, J. R. & Ram-Mohan, L. R. Band parameters for III-V compound semiconductors and their alloys. *J. Appl. Phys.* **89**, 5815–5875 (2001).

[9] Slater, J. C. & Koster, G. F. Simplified LCAO method for the periodic potential problem. *Phys. Rev.* **94**, 1498–1524 (1954).

[10] Chadi, D. J. Spin-orbit splitting in crystalline and compositionally disordered semiconductors. *Phys. Rev. B* **16**, 790–796 (1977).

[11] Harrison, W. A. *Electronic Structure and the Properties of Solids* (Freeman, San Francisco, 1980).

[12] Slater, J. C. Atomic shielding constants. *Phys. Rev.* **36**, 57–64 (1930).

[13] Clementi, E. & Raimondi, D. L. Atomic screening constants from SCF functions. *J. Chem. Phys.* **38**, 2686–2689 (1963).

[14] Tang, J.-M. & Flatté, M. E. Spin-orientation-dependent spatial structure of a magnetic acceptor state in a zinc-blende semiconductor. *Phys. Rev. B* **72**, 161315 (2005).

[15] Yakunin, A. M. *et al.* Spatial structure of Mn-Mn acceptor pairs in GaAs. *Phys. Rev. Lett.* **95**, 256402 (2005).

[16] Tang, J.-M. & Flatté, M. E. Multiband tight-binding model of local magnetism in $Ga_{1-x}Mn_xAs$. *Phys. Rev. Lett.* **92**, 047201 (2004).

[17] Kitchen, D., Richardella, A., Tang, J.-M., Flatté, M. E. & Yazdani, A. Atom-by-atom substitution of Mn in GaAs and visualization of their hole-mediated interactions. *Nature* **442**, 436–439 (2006).
